# Effects of pore size, water content, and oxygen-containing functional groups on oxygen adsorption in bituminous coal

**DOI:** 10.1038/s41598-023-37632-w

**Published:** 2023-06-26

**Authors:** Zhongjiu Ren, Dapeng Wang, Zheng Qin, Ziwen Liu

**Affiliations:** 1 China Coal Technology and Engineering Group Shenyang Research Institute, Fushun, 113122 China; 2 State Key Laboratory of Coal Mine Safety Technology, Fushun, 113122 China; 3 Shanxi Jinshen Energy Co. LTD, Xinzhou, 036500 China

**Keywords:** Energy, Organic chemistry, Physical chemistry, Environmental social sciences

## Abstract

To further explore the mechanism of coal spontaneous combustion and better grasp the laws of spontaneous combustion, this article studied the adsorption behavior of O_2_ in coal. Materials studio software was applied to study the adsorption of oxygen under different water content, different pore sizes, and different oxygen-containing functional groups by means of grand canonical Monte Carlo and molecular dynamics simulation methods. The results show that the adsorption capacity of O_2_ decreases with the increase in water content. With the increase of molecular pore size of coal, the adsorption capacity of O_2_ increases, and the tight adsorption amounts decrease. The equivalent adsorption heat is less than 42 kJ/mol, indicating that the adsorption of O_2_ in coal pores is physical adsorption. The smaller the physical adsorption energy and charge transfer value of the hydroxyl group for O_2_, it indicates that the hydroxyl group is the active group for the physical adsorption of O_2_.

## Introduction

Spontaneous combustion of coal not only wastes a large number of resources, but also damages the surrounding ecological environment, aggravates air pollution, and threatens the lives of relevant personnel^1–3^. Therefore, accurate and scientific determination of coal spontaneous combustion is of great significance to society and the public. However, coal is a substance with a complex structure and composition, and the oxidation of coal is accompanied by physical and chemical reactions^4–6^. The research on the low-temperature oxidation mechanism of coal is very important for proposing effective prevention and control measures, improving the efficiency of spontaneous combustion prevention, and has important practical value for protecting the personal safety of miners, protecting coal mine resources, and purifying the ecological environment^7–9^.

The low-temperature oxidation of coal is a complex process, and the factors affecting its oxidation include coal rank, pore size, temperature, and air humidity. Oxygen acts on the surface of coal through physical or chemical adsorption. In recent years, scholars have applied molecular mechanics, molecular dynamics, and Monte Carlo methods to the calculation of coal-adsorbed gases^10–12^. Sang et al.^13^ conducted in-depth research on the mechanism of interaction between coal and adsorbed gas, and proposed that in a solid gas adsorption system, the adsorption capacity plays a decisive role in the adsorption amount. Dai et al.^14^ proposed through research that the decisive factor for low-temperature oxidation of coal is its own oxygen absorption capacity; The three stages of physical adsorption, chemical adsorption, and chemical reaction occur simultaneously. Many scholars^15–18^ have conducted experimental research and theoretical analysis on the physical adsorption of oxygen by coal using coal spontaneous combustion tendency meters. A series of results indicate that factors affecting oxygen uptake include temperature, particle size, moisture, etc. Coal adsorption of oxygen can reach saturation in a short period of time, but the oxidation process is very slow; The premise of the coal spontaneous combustion process is the physical adsorption of coal oxygen, which mainly serves to transport oxygen for subsequent chemical adsorption and reaction. These studies often use the measurement of the amount of gas released by low-temperature oxidation of coal to calculate oxygen adsorption, which has certain limitations.

The coal-oxygen recombination reaction begins with the physical adsorption of coal and oxygen. After coal is in contact with oxygen, oxygen enters into the pore structure of coal, forming adsorption state on the coal surface, and further coal-oxygen composite reaction occurs. The pore structure of coal provides reaction space for coal-oxygen adsorption and composite reaction. At present, the research focus of coal adsorption gas is the adsorption of coalbed methane and gas, and the main research content is coalbed methane mining, gas extraction, etc. To further explore the mechanism of coal spontaneous combustion and better grasp the law of spontaneous combustion, we should increase the research on coal oxygen adsorption. In this paper, the process of coal physical adsorption of oxygen will be numerically simulated, analyze the relevant factors affecting coal oxygen adsorption, get the law of coal physical adsorption of oxygen, to provide theoretical support for the determination of coal spontaneous combustion, so as to better prevent accidents caused by coal spontaneous combustion.

## Methods

### Model construction and optimization

The molecular structure model of bituminous coal proposed by Li was adopted in this study^19^, whose molecular formula is C_174_H_148_O_5_N_2_.

**Figure 1:**
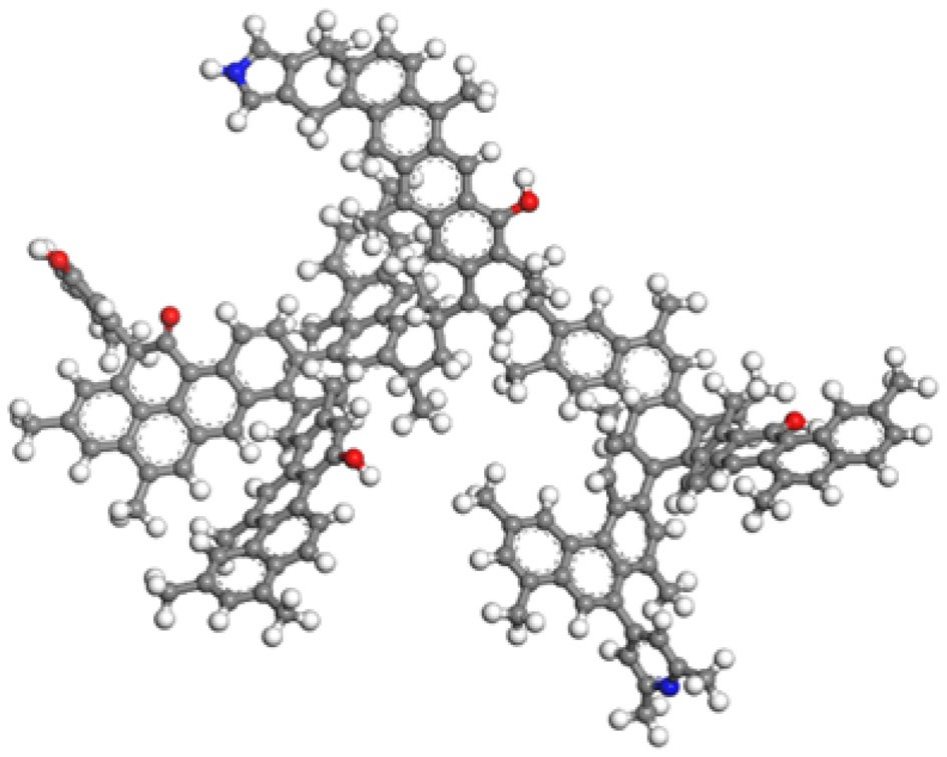
Single-cell stable configuration of coal molecules.

In order to obtain a relatively stable adsorbent structure model, this study carried out geometric optimization of coal molecular monolith under the Forcite module. The selection of the force field is very important in simulation calculation. In setting the force field, the Universal force field (UFF) was used. Compared with the Dreiding force field adopted by Sun et al.^20^, UFF includes all elements in the periodic table and can specify a method for charge calculation with high calculation accuracy. Smart flexible optimization method was adopted. The convergence mass deviation was set to Ultra-fine, the value 0.001 kcal/mol, the RMS Force 0.1 kcal/mol, and the RMS Displacement was set to 0.03 Å. The charge balance method was adopted for charge calculation, the cutoff radius was 4.5 Å, buffer width was 0.5 Å. Atom-Based van der Waals action was adopted, electrostatic action was the Ewald method, and the number of iterative calculation steps was set to 5000^21,22^. The stable configuration of the coal molecular cell obtained after the repeated iterative calculation is shown in Fig. 1.

After it obtained the constructed coal macromolecular models, it used the Forcite module to optimize the system at the steepest descent, eliminating overlapping conformations, and setting the maximum iterative steps, force field, charge, electrostatic, and van der Waals parameters as well as those specified in the optimization. Finally, the model of coal macromolecular surface adsorbent was optimized. In addition, the energy comparison before and after optimization of the model structure in Table 1 shows that the overall energy of the model decreases, indicating that the overall structure of the model tends to be stable and the stable configuration is finally obtained.

**Table 1:** Energy comparison before and after model structure optimization.

Energy	Pre-optimization energy/kJ/mol	Optimized energy/kJ/mol
Van der Waals	− 67.86	− 44.27
Total energy	− 295.91	− 117.22

### Adsorption simulation parameter settings

The GCMC method is used for adsorption simulation calculation, which is mainly used to solve the problem of molecular random diffusion, and is widely used in materials, chemistry, and physics^23^.

It is assumed that the molecular model of coal does not deform during the adsorption process and the layer spacing is unchanged. The adsorption of O_2_ was calculated under the Sorption module. Fixed pressure was selected for the task, and the Metropolis method was adopted. The simulated balance steps and process steps were both set as 1.5 × 10^6^ steps, the fixed pressure was 101 kPa, and the force field was the universal force field (UFF). The charge balance method (Qeq) was used for the charge, the Atom-Based van der Waals interaction was used, and the Ewald method was used for electrostatic interaction. The simulated adsorption calculation was carried out at a temperature of 298 K.

### Parameter setting of molecular dynamics simulation

After completing the adsorption calculation of the model, MD should be used to study the motion trajectories of molecules in the system, through which the adsorption concentration data can be obtained. The lowest energy configuration returned was selected, and the Dynamics task was selected under the Forcite module. The canonical ensemble (NVT) was used for simulation calculation, and the temperature corresponding to the adsorption temperature was selected respectively. The time step of the simulation was set as 1 fs, the total number of steps was 1 × 10^6^, and the total simulation time was 1000 ps. Other parameters were set with the adsorption simulation parameters. When researching the effect of water content on coal Adsorption of oxygen molecules, the first time to determine the adsorption position of water molecules in coal macromolecular structure model was using the adsorption module. After adding water molecules to the model, structural optimization and MD simulation need to be carried out again.

### Density functional theory

Density functional theory (DFT) calculation of the adsorption of different functional groups in coal molecules is carried out in the CASTEP (Cambridge sequential total energy package) module of MS software^24^. The exchange–correlation function for geometric optimization of different functional group structures uses the GGA-PWE function and the OBS method of PW91 functional (for dispersion correction), and the plane wave truncation energy is set to 400 eV^25^.

## Results and discussion

### Influence of moisture content on adsorption of O_2_

In this research, the O_2_ adsorption capacity of the coal structure model is calculated when the temperature is 298 K, the pressure is 101 kPa, and the water content is 0%, 0.5%, 1%, 2%, 3%, and 4% respectively. Corresponding to 0, 3, 5, 8, and 10 water molecules were added to the coal molecules.

**Figure 2:**
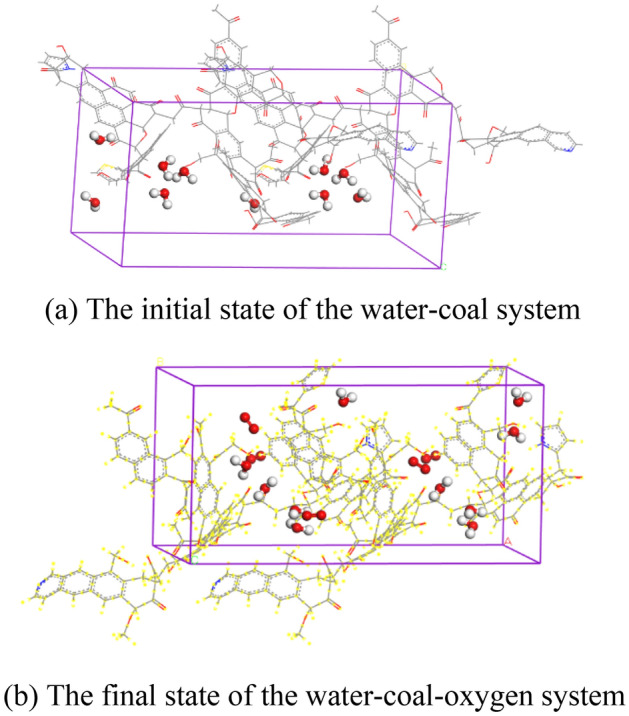
The initial and final states of the water-coal system and the water-coal-oxygen system.

Figure 2 is the initial and final states of the water-coal system and the water-coal-oxygen system.

The formula for calculating water content is as follows:

1 $$ W = \frac{{M_{{H_{2} O}} }}{{M{}_{{{\text{coal}}}} + M_{{H_{2} O}} }} \times 100\% $$

where M_H2O_ is the molecular mass of water, g/mol; M_coal_ is the molecular mass of coal, g/mol; W is water content, %.

**Figure 3:**
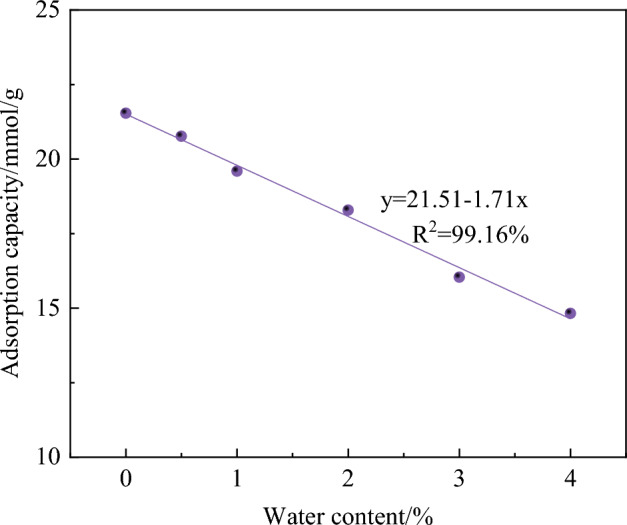
Relationship between adsorption capacity of O_2_ and moisture content.

When analyzing the influence of water content on gas adsorption capacity, the water content in coal is set as 0%, 0.5%, 1%, 2%, 3%, and 4% respectively. The relationship between oxygen adsorption capacity and water content is shown in Fig. 3. It can be seen from the figure that oxygen adsorption capacity decreases with the increase in water content. Through linear fitting, it is found that the adsorption capacity of O_2_ decreases linearly with the increase of water content in the range of 0–4%.

In the range of 0–4% water content, the adsorption capacity of O_2_ decreased from 21.54 mmol/g at 0% water content to 14.82 mmol/g at 4% water content. Water molecules are polar molecules. When dry coal is in contact with moist air, water molecules will react with oxygen-containing free radicals on the surface of coal to form chemically bound water. The free radical-oxygen-carbohydrate generated by the reaction promotes the formation of water and provides more active sites for the adsorption of coal and oxygen. The greater the amount of oxygen adsorbed by coal, the more water molecules in the air can promote the oxygen adsorption of coal at this stage. However, with the increasing water content, when all oxygen-containing functional groups are occupied, the excess water molecules begin to adsorb on the pore surface of coal in the form of free water and keep condensing, and finally form water-containing liquid film on the pore surface, hindering the diffusion and adsorption of oxygen^26^.

### Influence of pore size on oxygen adsorption

**Figure 4:**
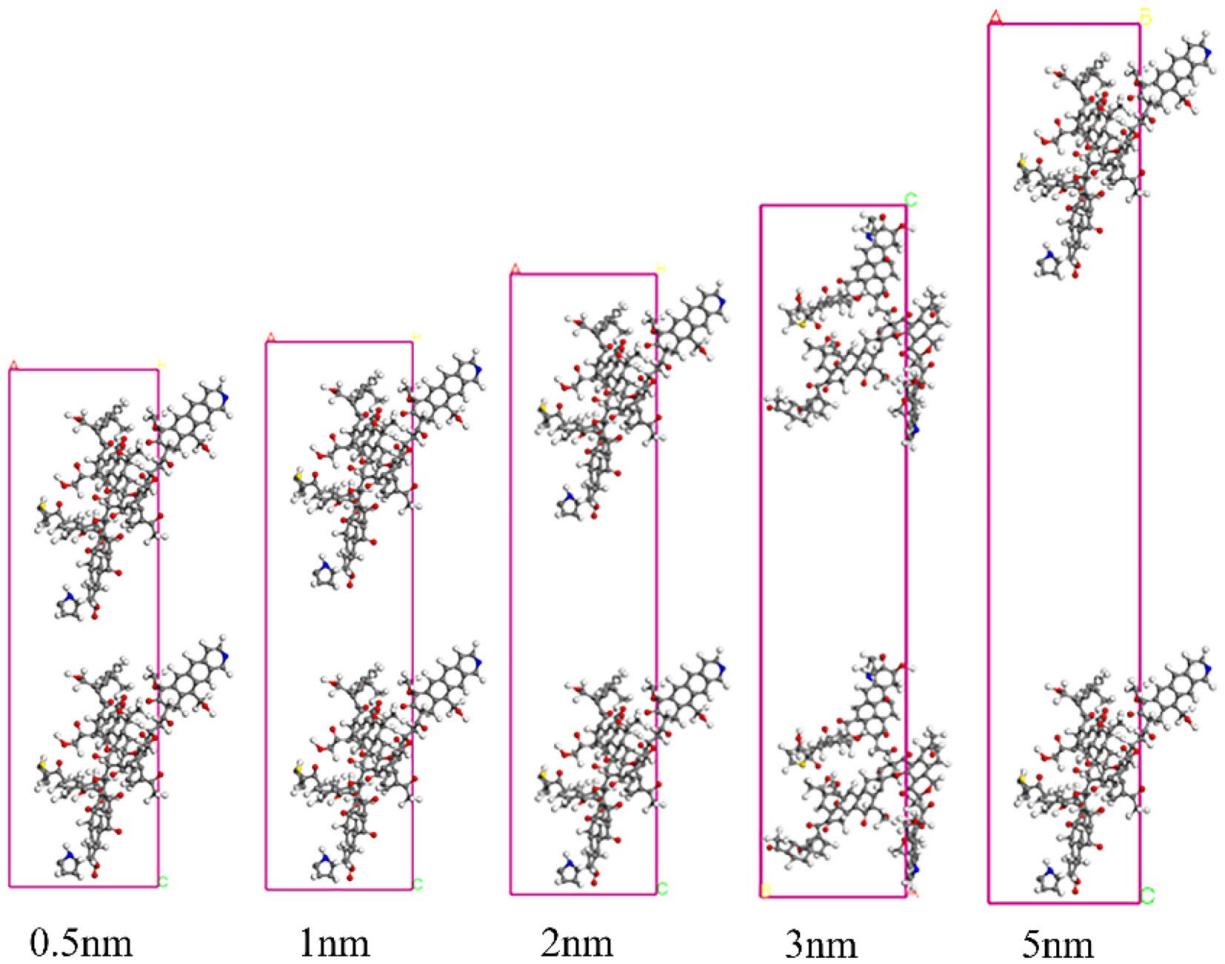
Different pore size structure models.

Coal is a porous medium consisting mainly of micropores and mesoporous. The micropore size is less than 2 nm and the mesopore size is between 2 and 50 nm. The adsorption behavior mainly occurred in micropores and mesoporous pores, while the diffusion behavior mainly occurred in mesoporous pores^27^. Figure 4 shows a model of the five types of pores.

**Figure 5:**
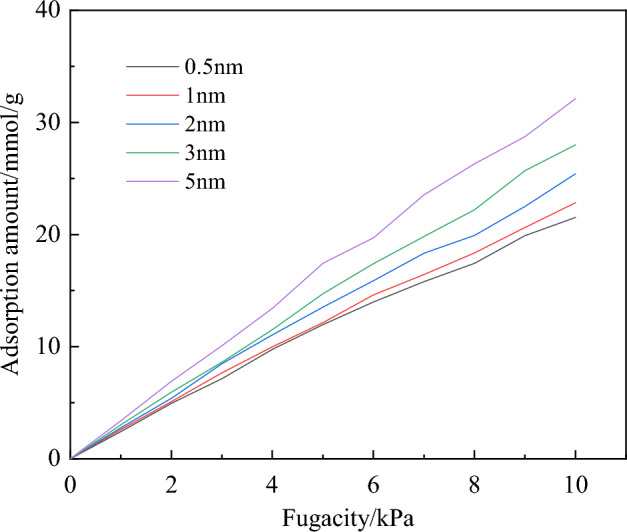
Isothermal loading curve of O_2_ within different pores.

As shown in Fig. 5, with the increase of the molecular pore size of coal, the adsorption capacity of gas also increases. This suggests that the larger the pore size, the more molecules of coal and gas can be accommodated in the pores. The adsorption heat of O_2_ ranges from 7 to 10 kJ/mol and is less than 42 kJ/mol, indicating that the adsorption of O_2_ in coal pores is physical adsorption^28^.

**Figure 6:**
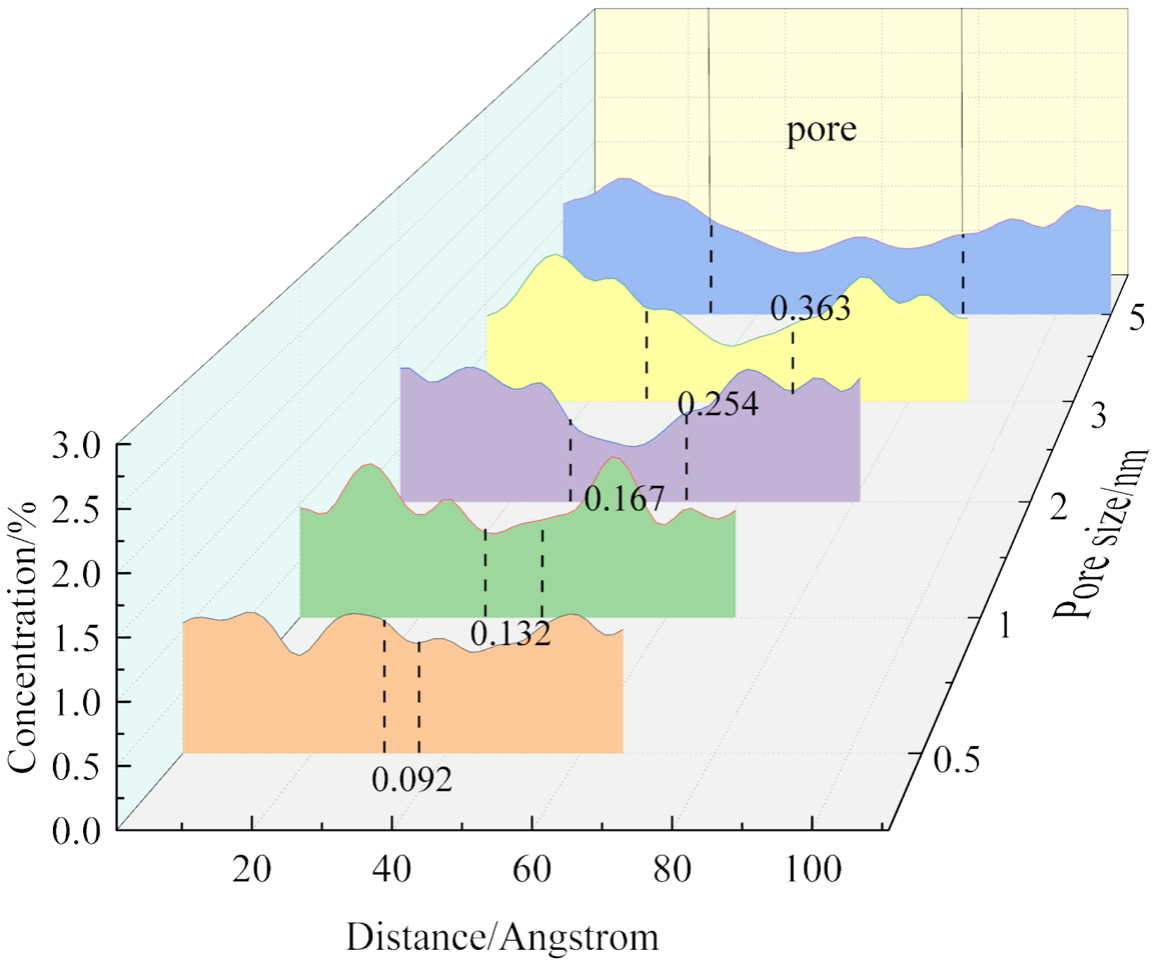
Gas concentration distribution under pores size.

By analyzing the concentration of gas molecules in different pore models, the distribution of gas molecules in the coal molecular layer and pores can be obtained, as shown in Fig. 6. When the pore size is 0.5 nm, the adsorption rate of O_2_ in the pore is 9.2%. It can be found that the concentration distribution of gas in the pore is proportional to the pore size.

**Figure 7:**
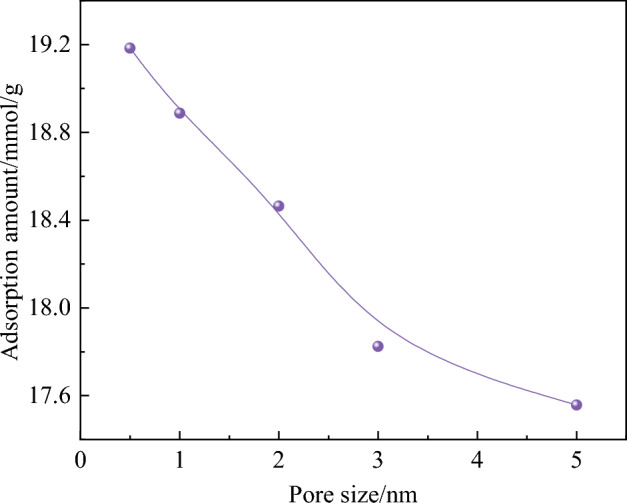
Tight gas adsorption capacity of different pore models.

The tight gas adsorption capacity of different pore models is shown in Fig. 7. The adsorption capacity of tight gas in different pore models decreases with the increase of pore size. In the 0.5 nm pore model, the adsorption capacity of O_2_ was 19.18 mmol/g. In the 5 nm pore model, the adsorption capacity of O_2_ is 17.55 mmol/g, which is 8.5% lower than that in the 0.5 nm pore model. In micropores, the distance between adjacent coal molecular layers is very small, so the coal molecular layer in micropores exerts greater force on gas molecules than the coal molecular layer in mesoporous pores. Therefore, the adsorption of gas molecules in coal molecules decreases with the increase of pore size. With the decrease in pore size, the number of pores exposed to air increased, and the oxygen adsorption capacity increased.

**Figure 8:**
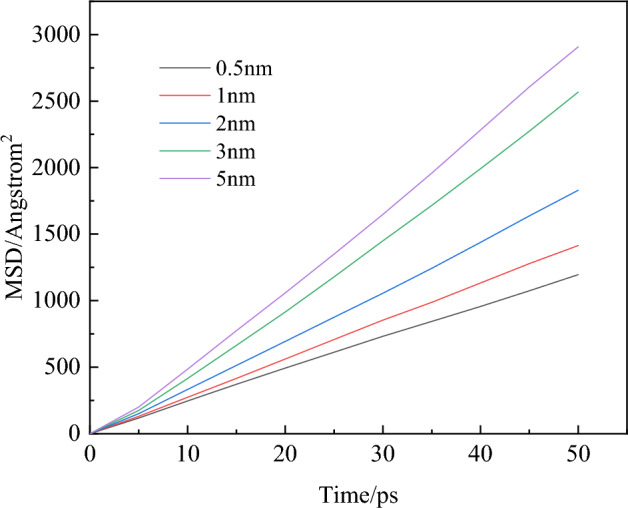
MSD of O_2_ within different pores.

The diffusion characteristics of gases with different pore sizes were studied. Through MD simulation, the relationship between the root mean square displacement (MSD) of the gas in the pore model and simulation time was obtained, as shown in Fig. 8.

**Figure 9:**
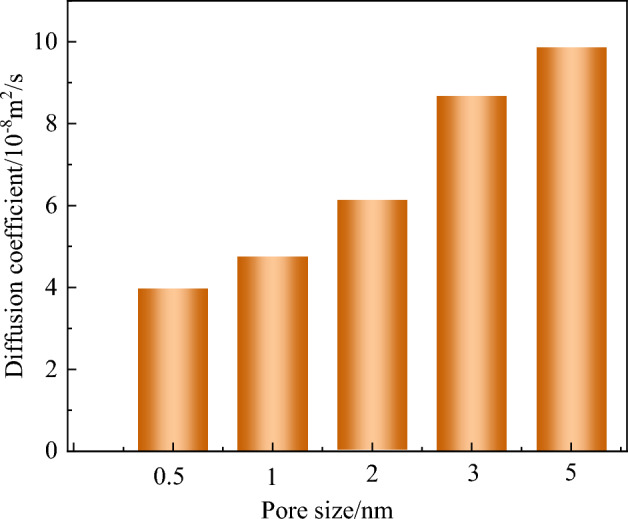
Gas diffusion coefficient within different pores.

The diffusion coefficient in Fig. 9 was obtained from the slope in Fig. 8. As shown in Fig. 9, the diffusion coefficient of the gas increases with the increase of the aperture. The diffusion coefficient of O_2_ increases from 3.98 × 10^−8^ to 9.85 × 10^−8^ m^2^/s. With the change of pore size, the gas diffusion in microporous structure is obviously weaker than that in mesoporous structure. In the effective cutoff radius (1.25 nm), gas molecules are more affected by van der Waals forces in the pore. In contrast, when the atomic spacing exceeds 1.25 nm, the effects of van der Waals forces and electrostatic forces are weakened and the diffusion of gas molecules through pores is enhanced^29^.

### Adsorption of O_2_ by different functional groups

**Figure 10:**
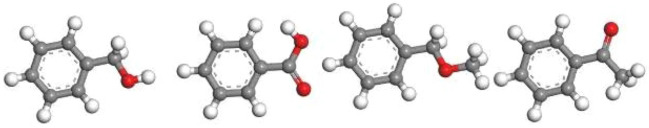
stable structure with different functional groups.

The carbon-containing functional groups on the surface of coal are mainly aromatic carbon (C–C) and lipid carbon (C–H), and the oxygen forms on the surface of coal are mainly carbonyl, hydroxyl, ether bond and carboxyl group. According to this result, four different coal structural units are constructed: –OH (phenolic hydroxyl unit), –COOH (carboxyl unit), –C=O (carbonyl unit,) and –O– (ether bond unit). The four different coal structural units and water substructure models are shown in Fig. 10. Four different coal structure units are optimized. Using the same exchange–correlation function, truncation energy, and convergence criteria as the bulk, the surface, O_2_ molecule, and four coal structural units are optimized geometrically. Based on quantum chemistry, the physical adsorption parameters of O_2_ adsorption by functional structural units on coal surface were calculated.

The adsorption energy of gas molecules on the surface of coal is defined as^30^:

2 $${\text{E}}_{{\rm ads}} = {\text{E}}_{{\rm coal/gas}}-{\text{E}}_{{\rm coal}}-{\text{E}}_{{\rm gas}}$$

In the formula, E_coal/gas_ is the total energy of coal adsorbed gas molecules; E_coal_ and E_gas_ are the energies of coal and gas molecules, respectively. According to this definition, E_ads_ is a negative value and represents exothermic adsorption. The larger the absolute value, the stronger the adsorption.

The physical adsorption parameters of O_2_ molecule are listed in Table 2. It can be seen that hydroxyl and ether bonds have lower physical adsorption energy values, and they have higher physical adsorption capacity for O_2_ molecules. The physical adsorption equilibrium distance between hydroxyl group and O_2_ is relatively small, while the physical adsorption distance between other adsorption sites and O_2_ is greater than 3 Å. Milliken charge transfer represents the degree of polarization of O_2_ in physical adsorption, with the hydroxyl group having a higher charge, followed by the carbonyl group and ether bond, indicating that O_2_ is adsorped more stably near these adsorption sites. During the adsorption process, the smaller the physical adsorption energy and charge transfer value of O_2_, indicating that O_2_ is more likely to be physically adsorbed around the adsorption site. Therefore, the hydroxyl group is the active group for the physical adsorption of O_2_.

**Table 2:** Adsorption parameters of molecular O_2_ at different adsorption sites.

Adsorption site	Adsorption energy /kcal/mol	Balance distance/Å	Charge transfer/e
–OH	− 2.987	2.324	− 0.040
–C–O–C	− 2.889	3.301	− 0.016
–C=O	− 2.336	3.174	− 0.030
–COOH	− 2.098	3.093	− 0.011

## Conclusion

MS software was applied to study the adsorption of oxygen under different water content, different pore sizes, and different oxygen-containing functional groups by means of GCMC and MD simulation methods. The conclusions are as follows:

1. With the increase in water content, oxygen adsorption capacity decreases. Water molecules gather on the pore surface of coal to form a water film, which hinders the transport and adsorption of oxygen and reduces the amount of oxygen adsorption.
2. The pore size of coal also affects the adsorption capacity of oxygen, and as the pore size of coal molecules increases, the adsorption capacity of O_2_ increases. However, the dense adsorption capacity decreases with the increase in pore size. The adsorption heat of equal amounts is less than 42 kJ/mol, indicating that the adsorption of O_2_ in coal pores is physical adsorption.
3. Due to the minimum physical adsorption energy and charge transfer value of hydroxyl on O_2_, it indicates that hydroxyl is the active group for the physical adsorption of O_2_.
4. One of the physical adsorption functions of coal spontaneous combustion is to transport oxygen for oxidation reaction. The study of the influencing factors in the process of low-temperature oxidation can provide theoretical basis for the analysis of the process of low-temperature oxidation of coal and the prevention and control of spontaneous combustion of coal. In the future, on the basis of this research, we will study inert gases, inhibitors and other fireproof materials to reduce the physical adsorption of coal oxygen, more effectively reduce the low-temperature oxidation of coal and the occurrence of coal fire.

## Data Availability

The datasets used and/or analysed during the current study available from the corresponding author on reasonable request.
